# Cyclosporin A-loaded dissolving microneedles for dermatitis therapy: Development, characterisation and efficacy in a delayed-type hypersensitivity in vivo model

**DOI:** 10.1007/s13346-024-01542-9

**Published:** 2024-03-12

**Authors:** Miquel Martínez-Navarrete, Antonio José Guillot, Maria C. Lobita, María Carmen Recio, Rosa Giner, Juan Aparicio-Blanco, María Carmen Montesinos, Hélder A. Santos, Ana Melero

**Affiliations:** 1 Department of Pharmacy and Pharmaceutical Technology and Parasitology, University of Valencia, Ave. Vicent Andrés Estellés s/n, 46100 Burjassot, Valencia Spain; 2 Department of Biomaterials and Biomedical Technology, University Medical Center Groningen, University of Groningen, Ant. Deusinglaan 1, 9713 AV Groningen, The Netherlands; 3 Department of Pharmacology, University of Valencia, Ave. Vicent Andrés Estellés s/n, 46100 Burjassot, Valencia Spain; 4 Department of Pharmaceutics and Food Technology, Faculty of Pharmacy, Complutense University of Madrid, Plaza Ramón y Cajal s/n, 28040 Madrid, Spain; 5 Interuniversity Research Institute for Molecular Recognition and Technological Development (IDM), University of Valencia, Polytechnic University of Valencia, Valencia, Spain; 6 Drug Research Program, Division of Pharmaceutical Chemistry and Technology, Faculty of Pharmacy, University of Helsinki, FI-00014 Helsinki, Finland

**Keywords:** Cyclosporin A, Lipid vesicles, Dissolving microneedles, Skin delivery, Dermatitis, Skin inflammatory conditions

## Abstract

Several drugs can be used for treating inflammatory skin pathologies like dermatitis and psoriasis. However, for the management of chronic and long-term cases, topical administration is preferred over oral delivery since it prevents certain issues due to systemic side effects from occurring. Cyclosporin A (CsA) has been used for this purpose; however, its high molecular weight (1202 Da) restricts the diffusion through the skin structure. Here, we developed a nano-in-micro device combining lipid vesicles (LVs) and dissolving microneedle array patches (DMAPs) for targeted skin delivery. CsA-LVs allowed the effective incorporation of CsA in the hydrophilic DMAP matrix despite the hydrophobicity of the drug. Polymeric matrix composed of poly (vinyl alcohol) (5% w/v), poly (vinyl pyrrolidine) (15% w/v) and CsA-LV dispersion (10% v/v) led to the formation of CsA-LVs@DMAPs with adequate mechanical properties to penetrate the *stratum corneum* barrier. The safety and biocompatibility were ensured in an in vitro viability test using HaCaT keratinocytes and L929 fibroblast cell lines. Ex vivo permeability studies in a Franz-diffusion cell setup showed effective drug retention in the skin structure. Finally, CsA-LVs@DMAPs were challenged in an in vivo murine model of delayed-type hypersensitivity to corroborate their potential to ameliorate skin inflammatory conditions. Different findings like photon emission reduction in bioluminescence study, normalisation of histological damage and decrease of inflammatory cytokines point out the effectivity of CsA-LVs@DMAPs to treat these conditions. Overall, our study demonstrates that CsA-LVs@DMAPs can downregulate the skin inflammatory environment which paves the way for their clinical translation and their use as an alternative to corticosteroid-based therapies.

**Graphical Abstract:**

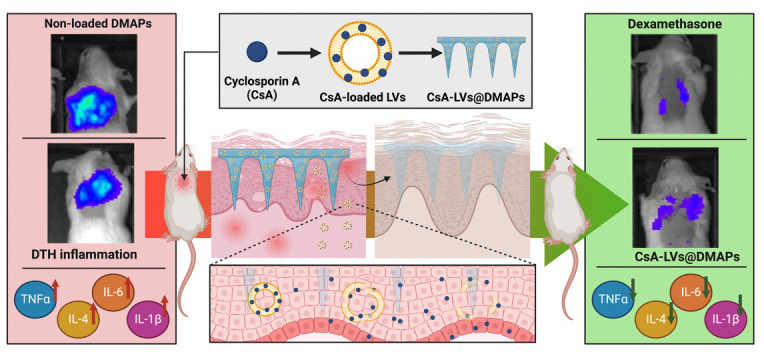

## Introduction

Atopic dermatitis (AD) is a chronic skin disease, which affects around 8% of the adult population and 20% of children worldwide. The main symptoms are eczematous eruptions and inflamed, red, dry or itchy skin lesions [1] and are associated with a wide board of comorbidities like asthma, allergies, eosinophilic oesophagitis, rhinitis, autoimmune diseases [2–4], depression and anxiety [5, 6]. All these symptoms, together with important economic burden [7], negatively affect the life quality of patients [8].

Topical corticosteroids (TCSs) constitute a usual first-line treatment for AD [9], despite their multiple local adverse effects, specifically when used in sustained therapies or on extensive skin areas [10]. TCSs are associated with skin thinning, telangiectasia, striae, purpura, focal hyperhidrosis, hypopigmentation and perioral dermatitis [11, 12]. Additionally, long-term exposure or multiple daily doses exacerbate the skin barrier disruption, increasing its systemic absorption, leading to rebound flares after treatment discontinuation [13, 14], adrenal suppression, poor growth, hypertension, hyperglycaemia, insulin resistance and cataracts [15–19]. Moreover, TCSs can be ineffective in moderate-to-severe cases of AD, where the oral administration of corticosteroids becomes the first choice, which frequently aggravates the side effects [20].

Alternative therapeutic strategies like phototherapy or administration of systemic immunosuppressants (cyclosporin, methotrexate, mycophenolate mofetil or azathioprine) are also available; however, they are associated with even more serious side effects [9]. Therefore, their use is restricted to severe AD cases in which the life quality of the patient is strongly affected [21–23]. Among them, oral cyclosporin A (CsA) is of special interest due to the good response observed in most patients. However, the severity of the adverse events, such as the increased risk of infection, nephrotoxicity, hypertension, tremor, hypertrichosis, headache, gingival hyperplasia, increased risk of skin cancer and lymphoma and renal dysfunction, limits its use in cycles, having drug-free periods between flares [24–26]. Therefore, topical CsA administration directly to the target tissue could minimise or even avoid the off-target side effects, thus becoming an alternative treatment to long-term topical corticoids [27, 28].

The skin barrier function of the *stratum corneum* (SC) limits the free diffusion of molecules > 500 Da, which is the case of CsA (1200 Da) [29]. A wide variety of skin permeability-enhancing techniques has been used to increase drug permeability through the skin [28, 30–32]. Several nano-sized particles, like flexosomes, have proved their efficacy in delivering CsA topically [33–35]. However, it seems that these vesicles do not intactly cross the skin barrier and merge within the skin lipids, contributing to a disruption of the barrier function [36]. Microneedle array patches (MAPs) stand out as non-invasive and efficient strategies to increase drug delivery through the skin [37, 38]. Moreover, they are also compatible with a wide variety of nanoparticles [39–41]. MAPs are devices that contain micro-sized needle-like projections that can mechanically disrupt the SC barrier and reach the deeper skin layers without reaching the nerve endings of the skin [42, 43]. Depending on the manufacturing materials and the approach used, MAPs can achieve different goals such as vaccination [44–46], systemic drug delivery [47–49], local drug delivery [50] or sustained drug release [51, 52].

Herein, we report a combined strategy coupling lipid-based vesicles and MAPs to achieve a local CsA delivery into the skin, aiming for local therapy of skin inflammation. Poly(vinyl alcohol) (PVA) and poly(vinyl pyrrolidone) (PVP) were used to produce dissolving MAPs (DMAPs), which release the cargo in a controlled manner when the polymers dissolve in the interstitial skin fluids. CsA was previously incorporated into nano-sized liposomes to increase the cargo loading in the hydrophilic DMAP matrix. The manufactured CsA-LVs@DMAPs were characterised in terms of morphology and mechanical properties. The efficacy of the developed formulation was studied in an ex vivo setup, and its biocompatibility was tested in vitro in both fibroblast and keratinocyte cell lines. Finally, the efficacy of CsA-LVs@DMAPs to ameliorate skin inflammation was studied in vivo in a delayed-type hypersensitivity murine model.

## Materials and methods

### Materials

Phospholipon 90G (P90G) was purchased from Lipoid (Steinhausen, Switzerland). CsA (purity > 99%), poly-(vinyl alcohol) (PVA) (Mw = 9–10 kDa, 80% hydrolysed), sodium dodecyl sulphate (SDS), 3-aminophtalhydrazinde monosodium salt (luminol, purity > 98%), oxazolone (purity > 99%) and ammonium molybdate (purity > 99%) were purchased from Sigma-Aldrich (St. Louis, USA). Polysorbate 80 (Tween^®^ 80) and L-( +)-ascorbic acid (purity > 99%) were purchased from Scharlab (Sentmenat, Spain). HPLC-quality methanol (MeOH), ethanol (EtOH) and acetonitrile (ACN) were obtained from Sigma-Aldrich (St. Louis, USA). Acetone (purity 99.9%) was purchased from ACROS Organics (Geel, Belgium). Ultrapure water (UPW) was obtained by a Milli-Q purification system with resistance > 18 MΩ cm and TOC < 10 ppb. Poly-(vinyl pyrrolidone) (PVP) (Mw = 40 kDa) was acquired from Tokyo Chemical Industry (Tokyo, Japan). L-( +)-Ascorbic acid (purity > 99%) was purchased from Scharlab (Sentmenat, Spain). TNF-α, IL-4, IL-1β and IL-6 ELISA kits were acquired from ThermoFisher Scientific (Waltham, USA).

### Methods

#### Analytical determination and quantification of CsA by high-performance liquid chromatography

CsA concentrations were quantified by high-performance liquid chromatography (HPLC) (Agilent 1260 Infinity II; Agilent, Santa Clara, USA). The mobile phase consisted of a mixture of EtOH-to-UPW-to-ACN (65:30:5) delivered with a flow of 1 mL/min. A reverse-phase column C18 (Brisa LC^2^ C18 5 µm, 15 × 0.46 cm) was used as a stationary phase. The UV–Vis detector wavelength and the column oven temperature were set up at 210 nm and 55 °C, respectively. The analysis time of each sample was 8 min, and CsA time retention time was 6 min [53]. The analytical method was validated intraday and interday in terms of specificity, linearity, precision accuracy and robustness within the concentration range 0.2 − 100 µg/mL.

#### CsA-LV preparation

CsA-LVs were produced with the thin-film hydration method [34]. Briefly, P90G (60 mM), Tween^®^ 80 (5 mM) and CsA (1.5 mM) were dissolved in MeOH. Then, the organic solvent was distilled in a rotatory evaporator at a temperature of 55 °C and 250 bar for 12 h to remove the organic solvent traces (R-100; BUCHI Ibérica, Barcelona, Spain). The obtained thin-film was then reconstituted with 10 mL of UPW for 1 h at a temperature of 55 °C and sonicated for 2 h at 50 °C. To reduce the CsA-LVs, the dispersion was first filtered through 0.45- and 0.22-µm pore-size filters and then extruded through 200 nm polycarbonate membranes at 50 °C (20 times). To remove the excess of non-entrapped components, the CsA-LV dispersion was purified by dialysis against 2 L of phosphate-buffered saline (PBS) pH 7.4 at 4 °C for 24 h (Spectra/Por^®^ regenerated cellulose dialysis tubing, 14 MWCO; Repligen, Wattman, USA) [33].

#### CsA-LV characterisation

CsA-LVs were characterised in terms of size, polydispersity index (PDI), zeta (ζ)-potential, entrapment efficacy (EE) and the efficiency of the production process was evaluated by phospholipid recovery rate.

##### Size, PDI and ζ-potential

Particle size, PDI and ζ-potential were measured using Zetasizer Nano Series (Malvern Instruments, Malvern, UK). Dynamic light scattering (DLS) mode was used to measure vesicular size (average diameter) and PDI. Laser Doppler electrophoresis (LDE) was used to determine de ζ-potential [54]. All measurements were conducted at room temperature, and the reported results were obtained as an average of three different batches (*n* = 3).

##### Entrapment efficacy

CsA-LVs were disrupted using a lysis mixture of SDS 1% (w/v) solution in UPW and MeOH (55:45). One hundred microlitres of the liposomes was added to 900 µL of the lysis mixture and vortexed for 1 h. The CsA was then measured by HPLC. The reported results were obtained as an average of three different batches (*n* = 3). The entrapment efficiency (EE) was calculated as follows (Eq. 1) [55]:

1 $${\text{EE}}\;\left(\%\right)=\frac{Qe}{{Q}_{t}} \times 100$$

where *Q*_*e*_ is the amount quantified by HPLC after the vesicle disruption and *Q*_*t*_ is the total amount of CsA used for CsA-LV preparation.

##### Phospholipid recovery

Phospholipid content in the CsA-LV solution samples was determined following an adaptation of the Rouser et al. method [56]. For that, 100 µL of the CsA-LV formulation was added into Pyrex tubes and heated to 270 °C until solvent evaporation using a sand bath (OVAN SB400-E; Badalona, Spain). Next, 450 µL of HClO_4_ 70% (v/v) was added to the mixture and heated for 30 min at 250 °C. Once the samples were cooled down, 3.5 mL of UPW, 500 µL of ammonium molybdate 2.5% (w/v) and 500 µL of ascorbic acid 10% (w/v) were added, vortexed and incubated for 7 min at 100 °C. Next, the reaction was stopped by cooling down the samples in an ice bath. Finally, the samples were filtered, and the absorbance was measured with a UV–Vis spectrophotometer (U-2900 spectrophotometer; Hitachi, Tokyo, Japan). The reported results were obtained as an average of three different batches (*n* = 3).

##### CsA-LV morphology: TEM imaging

To visualise the morphology of CsA-LVs, they were stained using an adaptation of the Théry et al. method [57]. Briefly, the CsA-LVs (1:100 dilution) were fixed in paraformaldehyde 2% (w/v) for 30 min and then deposited on carbon-coated grids for 15 min. Next, samples were washed and fixed with glutaraldehyde 1% (w/v) for 5 min, washed again and then contrasted in uranyl acetate 1% (w/v) and methyl cellulose 0.5% (w/v). Samples were analysed with HT7800 transmission electron microscope (Hitachi, Tokyo, Japan). Images were recorded using a CMOS EMSIS XAROSA digital camera at 100 kV [58].

#### CsA-LVs@DMAP preparation

CsA-LVs@DMAPs were fabricated using the solvent-casting method in a one-step manufacturing process. For that, PVA and PVP aqueous polymeric blends were prepared and mixed with CsA-LVs for preparing different CsA-LVs@DMAP prototypes (Table 1). These polymer mixtures were then cast onto PDMS moulds—600 µm height, 200 µm base (Micropoint Technologies, Singapore)—and a positive pressure of 3–4 bar was applied for 40 min to fill the mould cavities. Afterwards, they were dried at room temperature for 2–3 days before demoulding and stored in a dry-seal desiccator [59].

**Table 1:** Composition of the three CsA-LVs@DMAP formulations developed

	**Formulation**		
**Components**	**F1**	**F2**	**F3**
PVA (% w/v)	20	–	10
PVP (% w/v)	–	10	5
CsA-LVs (% v/v)	10	10	10
dd-H_2_O	70	80	75

#### CsA-LVs@DMAP characterisation

CsA-LVs@DMAP mechanical properties were characterised in terms of morphology, residual water content, mechanical resistance, compression, insertion properties and drug release performance.

##### CsA-LVs@DMAP morphology: SEM imaging

CsA-LVs@DMAP 3D morphology was observed by scanning electron microscopy. For that, CsA-LVs@DMAPs were coated using the Co/Au layer. Images were recorded using a field electron scanning emission microscope (FESEM) SCIOS 2 FIB-SEM (ThermoFisher Scientific, Waltham, USA) at 20 kV. Length measures were performed using ImageJ software (NIH, Wisconsin, USA).

##### Thermogravimetric analysis

Thermogravimetric analysis (TGA) was performed on the CsA-LVs@DMAPs to determine the residual water content of the CsA-LVs@DMAPs as a measure of their stability after CsA-LVs@DMAP removal from PDMS moulds, 1 week and 6 weeks (TG 209 F3 Tarsus; NETZSCH, Waldkraiburg, Germany). Temperature scans were performed from 25 to 200 °C at a scan rate of 10 °C/min [60]. NETZSCH Proteus Thermal Analysis 8.0 software was used to create baselines and thermograms (NETZSCH, Waldkraiburg, Germany). The content of water was measured by the weight loss of the DMAPs from 0 to 100 °C.

##### In vitro drug release

In vitro drug release from CsA-LVs@DMAP formulations was performed using an adapted method from Larrañeta et al. [61]. In this methodology, one CsA-LVs@DMAP loaded with 40 µg of CsA was immersed into a 5 mL solution of PBS pH 7.4 warmed at 32 °C without stirring to release the CsA-LVs. At certain time points (0, 5, 10, 15, 20, 30, 45, 60 and 90 min), samples of 0.2 mL were taken, and 0.1 mL of MeOH was added to induce the CsA-LV disruption and CsA release. The CsA solubility in this medium was quantified by HPLC (1.2 mg/mL) in order to ensure the sink conditions throughout the experiment. Then, CsA was quantified by HPLC, and the cumulative drug amounts were calculated. Percentage of drug released was plotted versus time. The reported results were obtained as an average of three different batches (*n* = 3).

##### Deformation and insertion properties after compression in an artificial skin model

Mechanical strength and insertion properties of the different CsA-LVs@DMAPs were evaluated by their compression and penetration performance in an artificial skin model [62]. For that, 8 layers of Parafilm-M^®^ were disposed one above the other, simulating an artificial skin tissue, and the CsA-LVs@DMAPs were then applied onto the parafilm simulating a thumb-force insertion (32 N/array) and keeping pressure for 30 s. The number of holes created in each Parafilm-M^®^ layer was recorded, and the deformation of the CsA-LVs@DMAP tips after compression was measured under optical microscopy. Length measures were performed using ImageJ software (NIH, Wisconsin, USA). Compression percentage was calculated as described in Eq. 2 [63]:

2 $${\text{Deformation}}\:\left(\%\right)=\frac{{h}_{0}-{h}_{f}}{{h}_{0}}\times 100$$

where ${h}_{0}$ is the tip length before compression and ${h}_{f}$ is the tip length after compression. The reported results were obtained as an average of three different batches (*n* = 3).

#### In vitro biocompatibility in fibroblasts and keratinocytes cell lines

Cytotoxicity was evaluated using L929 fibroblasts (passages 55–60) and HaCaT keratinocytes (passages 51–54) [64]. Both cell types were cultured in separated flasks (75 cm^2^) in Modified Eagle’s Medium (MEM) supplemented with 1% (v/v) GlutaMax™ (Gibco, Invitrogen, USA) and Dulbecco’s Modified Eagle’s Medium high glucose (4.5 g/L) (HyClone, Logan, UT) respectively, containing both 10% of foetal bovine serum (Gibco, Invitrogen, USA), 1% (v/v) of L-glutamine, 1% (v/v) of nonessential amino acids, 100 IU/mL of penicillin and 100 mg/mL of streptomycin (HyClone, Logan, UT). Cells were cultured at 37 °C, 5% CO_2_ and 95% relative humidity. The medium changed every other day, and before reaching the confluence (60–70% confluence), cells were detached using trypsin-to-EDTA (0.25%).

Prior to each test, cells were harvested and diluted at a density of 1 × 10^5^ cells/mL. The cells were then seeded in separated 96-well plates for 24 h (0.2 mL per well). Then, the medium was discarded, and cells were washed with 200 μL of PBS pH 7.4. CsA-LVs were diluted and added to each well at a concentration of 25, 100 and 250 μg/mL and incubated for 24 h. Cells were also exposed to dissolved CsA-LVs@DMAPs for 24 h. Cells were washed twice with PBS pH 7.4, and the number of viable cells was quantified using the CellTiter-Glo^®^ assay according to the manufacturer’s instructions [65]. For that, 10 μL of CellTiter-Glo^®^ reagent and 10 μL of PBS pH 7.4 were added to each well. Luminescence was measured using a Synergy H1 microplate reader (BioTek, Vermont, USA). SDS 1% and untreated cells were used as positive and negative controls, respectively. The reported results were obtained as an average of three independent assays (*n* = 3).

#### Ex vivo skin permeability

CsA-LV and CsA-LVs@DMAP permeability were tested ex vivo using a Franz-diffusion Cell (FDC) setup using full-thickness mice skin, which was placed horizontally between donor and receptor chambers with the *stratum corneum* facing upwards (effective diffusion area of 1.76 cm^2^). The donor chamber was filled with 0.5 mL of CsA-LV dispersion or one CsA-LVs@DMAP inserted into the skin using a syringe plunger to apply a firm pressure for 30 s. The receptor chamber was filled with 12 mL of receptor media PBS pH 7.4-to-EtOH (1:1) maintained at 32 ± 1 °C under stirring throughout the whole experiment. Before starting, the CsA solubility in the receptor media was measured by HPLC (10 mg/mL) to guarantee that sink conditions were maintained throughout the experiment. Samples of 200 µL were collected at 1.5, 3, 12, 24 and 48 h (*n* = 6), and the sample volume was replaced with a pre-warmed receptor medium at each sampling time. Furthermore, the CsA retention in the skin structure was determined after 2, 6, 16, 24 and 48 h. For that, the skin was removed from the FDC setup, cut into small pieces and incubated in MeOH for 24 h under agitation to extract the CsA (*n* = 3). All samples were filtered prior to CsA quantification by HPLC, and the cumulative amounts of the drug were plotted versus time [66].

#### Ex vivo CsA-LVs@DMAP skin insertion performance and dissolution time

CsA-LVs@DMAPs were observed by optical microscopy and then inserted in full-thickness mice skin with thumb force and keeping pressure for 30 s. They were removed after 2, 6, 12 and 24 h post-insertion and imaged. Additionally, CsA-LVs@DMAPs were co-loaded with methylene blue (2% w/v) to observe the cargo deposition within the skin structure after 2, 6, 12 and 24 h post-insertion (Juision 2 K camera).

#### In vivo delayed-type hypersensitivity murine model

**Fig. 1:**
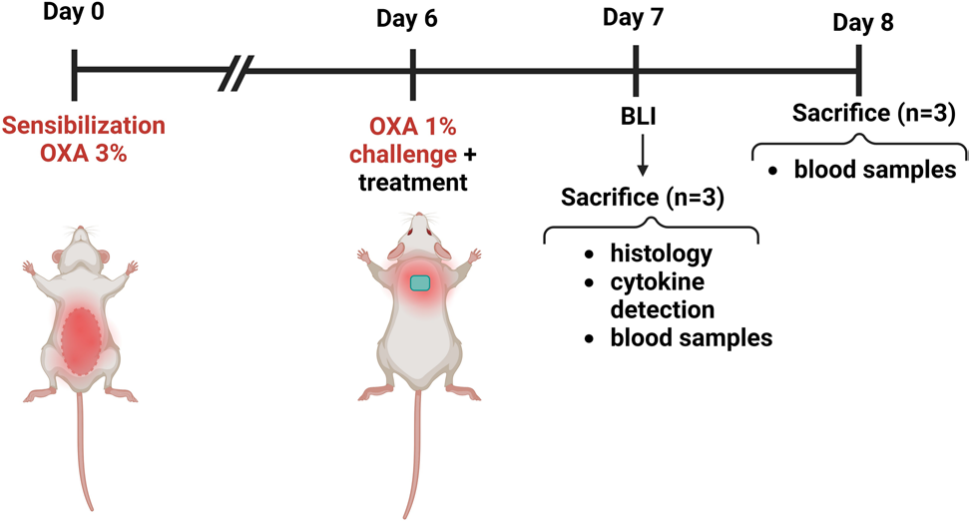
Schematic illustration of the delayed-type hypersensitivity (DTH) murine in vivo model

For the in vivo experiments, swiss-CD1 female mice (4–6 weeks old) were acquired from Harlan Interfauna Iberica (Barcelona, Spain) and stabled in SCSIE facilities (University of Valencia). Housing conditions and all in vivo procedures were approved by the Institutional Ethics Committee of the University of Valencia and the regional government in accordance with the guidelines established by the European Union on Animal Care (2023-VSC-PEA-0064). Figure 1 summarises the timeline of the delayed-type hypersensitivity (DTH) in vivo model carried out.

##### Induction of the delayed-type hypersensitivity

Briefly, on day 0, abdominal areas were shaved and sensitised with a single topical application of 150 µL oxazolone (OXA) (3% w/v) dissolved in acetone. On day 6, the backs of the mice were shaved and challenged with a second administration of 150 µL of OXA (1% w/v). Finally, the treatments were applied. Mice were randomly divided into five different groups prior to the experiments divided as follows: (1) healthy control group (unchallenged), (2) non-treated group after OXA challenge (OXA), (3) group treated with dexamethasone (DEXA) after OXA challenge—as a reference TCS treatment topically applied in the back (150 µL of DEXA dissolved in EtOH-to-UPW 7:3, 0.2 mg/µL)—(4) group treated with CsA-LVs@DMAPs after OXA challenge and (5) group treated with blank DMAPs (without CsA-LV loading) after OXA challenge. In case of DMAPs, the skin was hydrated with PBS pH 7.4 before the insertion, and then, DMAPs were secured to the skin using physiotherapy tape and stitches [67].

##### Bioluminescence imaging

Bioluminescence imaging (BLI) was performed using an IVIS Lumina X5 bioluminescence imaging system (PerkinElmer, Waltham, USA). Images were recorded 24 h after the OXA challenge. For this, mice received an intraperitoneal injection of luminol sodium salt (200 mg/kg) and were then anaesthetized by isoflurane inhalation. Imaging was performed 20 min after luminol administration. The imaging conditions were an exposure time of 60 s and binning 8. Regions of interest (ROI) were selected on the CsA-LVs@DMAP administration sites, and the total photon emission was measured (*n* = 6) [67]. After the BLI study, mice were killed by cervical dislocation under optimal anaesthesia and analgesia conditions.

##### CsA plasma level quantification

Blood samples were taken from all mice groups at the final point by cardiac puncture at 24 or 48 h after the OXA challenge. Blood samples were kept in heparinised vials, then deproteinised using MeOH (blood-to-MeOH 1:4) and centrifuged at 6100 × *g* for 10 min at 8 °C (Centurion 8000 series; Centurion Scientific Global, Chichester, UK) [68]. Plasma samples were analysed by HPLC. The reported results were obtained as an average of three different mice (*n* = 3).

##### Histological study

Skin biopsies were obtained from the back tissues using a biopsy punch and carefully divided for further studies. For the histological study, they were first fixed in paraformaldehyde 4% (w/v). Next, samples were washed, dehydrated, paraffin-embedded and cut into 10-µm-thick sections. The samples were stained using haematoxylin-eosin (H&E) and the Masson trichrome (MT) collagen stain. Epidermal and collagen deposition thickness was measured using ImageJ software (NIH, Wisconsin, USA) in × 20 magnification images [69]. From each group, 3 samples from 3 different mice were initially analysed by a blinded investigator.

##### Inflammatory cytokine detection and myeloperoxidase activity quantification

Skin samples were homogenised by freezing them with liquid nitrogen, pulverised and embedded in a lysis buffer as described previously [70]. For enzyme-linked immunosorbent assays (ELISA), commercial kits were used to quantify IL-1β, IL-6, TNF-α and IL-4 as inflammatory mediators. Quantification was done according to the instructions provided by the ELISA kit manufacturer (ThermoFisher Scientific, Waltham, USA). For the myeloperoxidase (MPO) quantification, 5 µL of tissue homogenates, 195 µL of PBS pH 7.4, 20 µL of phosphate-buffered saline pH 5.4, 20 µL of hydrogen peroxide 0.052% (w/v) and 20 µL of tetramethylbenzidine (TMB) 15 mM on dimethylformamide (DMF) 8% (w/v) were mixed and incubated at 37 °C for 10 min [70]. The reaction was stopped with 100 µL of sulphuric acid 2N, and absorbance was measured at 450 nm with a plate reader (VICTOR^3^ Multilabel Plate Reader; PerkinElmer, Waltham, USA). The reported results were obtained as an average of three different mice (*n* = 3).

#### Statistical analysis

Data processing was performed using Microsoft Excel 2016^®^ (Redmond, WA, USA) and SPSS version 22.0^®^ (IBM Corp., NY, USA). Data are expressed as the mean ± standard deviation (SD) unless otherwise stated. Statistical analysis was carried out using Student’s *t*-test for simple comparisons and one-way ANOVA followed by the Tukey post hoc test. Assumptions of normality and homogeneity of variances were considered in all analyses.

## Results and discussion

### CsA-LV characterisation

**Fig. 2:**
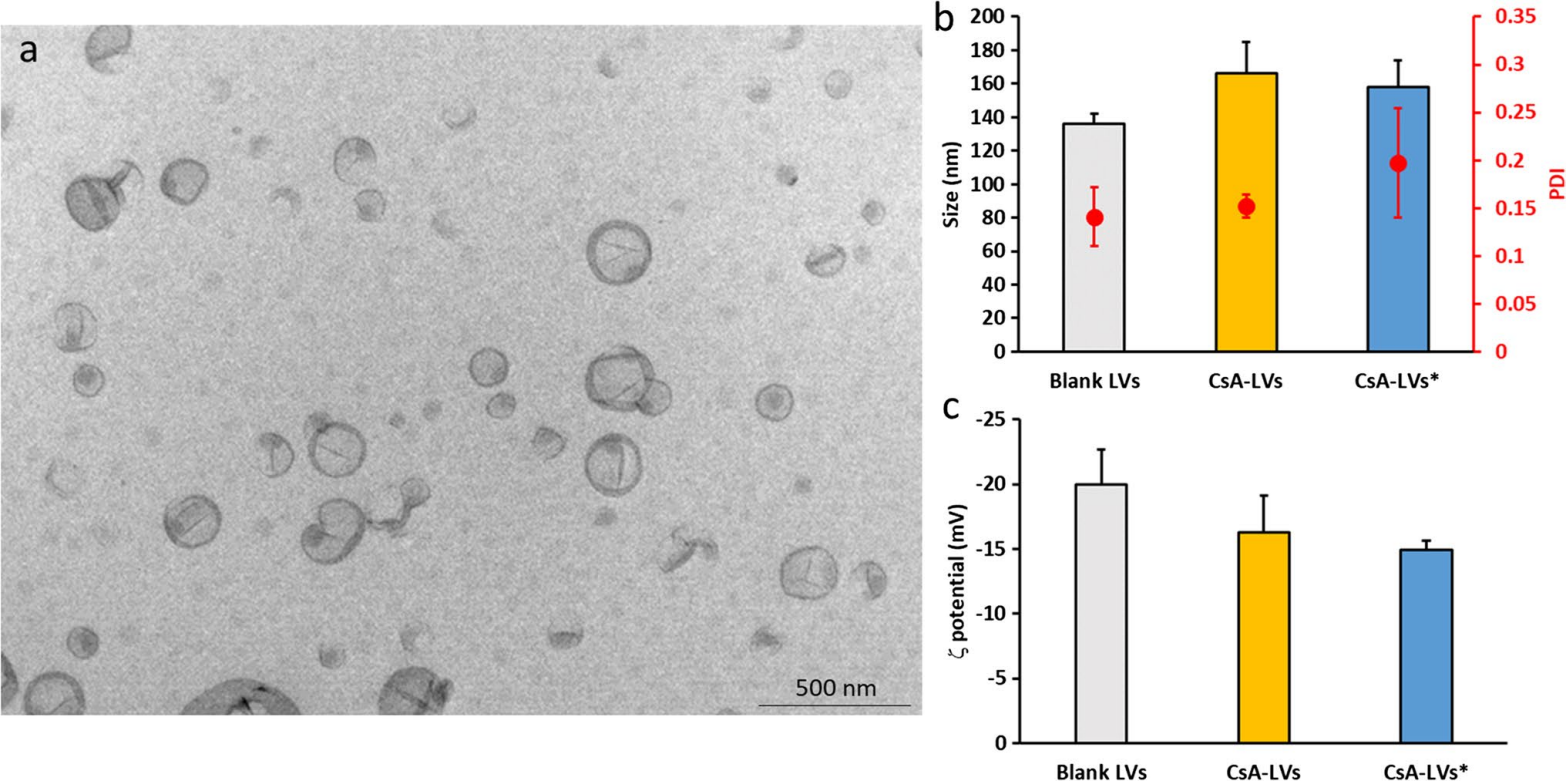
**a** Representative TEM image of CsA-LVs. **b** Size and PDI of the blank LVs and CsA-LVs. Results are expressed as mean ± SD (*n* = 3). **c** ζ-potential of the blank LVs and CsA-LVs. Results are expressed as mean ± SD (*n* = 3). No statistical differences were found between CsA-LVs and blank LVs. * denotes that the measurement has been performed after dissolving CsA-LVs@DMAPs for releasing the CsA-LVs in aqueous media

CsA-LVs were successfully produced by the thin-film hydration method [71]. After the hydration of the thin film, a milky suspension of lipid vesicles was obtained. The phospholipid recovery obtained indicates an optimal yield of the fabrication method (93.2 ± 1.7%). As observed in Fig. 2a by TEM imaging, a homogeneous round-like lipid vesicle population was formed, as previously reported [72]. The DLS measurements of PDI (0.152 ± 0.012) confirmed the optimal homogeneity of the sample for pharmaceutical purposes, since it has been reported that values lower than 0.3 are optimal for skin drug delivery [73]. Moreover, the CsA-LVs have an adequate size of 166 ± 19 nm (Fig. 2b) [74, 75]. The mean diameter of the CsA-LVs is in concordance with other studies. For instance, Hinna et al.’s study reported similar characterisation results of lipid vesicles produced with the same fabrication method and sonication/extrusion size reduction process [76]. In addition, no differences were observed between the CsA-loaded LVs and non-loaded LVs regarding PDI and size, showing values of 0.141 ± 0.031 and 136 ± 6 nm, respectively (*p* > 0.05). CsA-LVs exhibited a negative ζ-potential (− 16.3 ± 2.7 mV) (Fig. 2c), which is in correlation with other works that used DPPC and Tween^®^ 80 in their lipid-based formulations [33, 77, 78]. However, the CsA-LVs produced in this work showed a discrete negative value, probably caused by the zwitterionic behaviour of the lipid used in the formulation (DPPC) [79]. Although high ζ-potential values are related with an improved long-term stability, they have also been related to higher cellular uptake rates [80], which could lead to undesirable toxicity rates.

As a hydrophobic drug, CsA cannot be easily loaded into a hydrophilic PVP-PVA polymeric matrix; however, LVs allow the incorporation of such kinds of drugs into them. Nevertheless, a high loading capacity is crucial for this purpose, since the loading of LVs in DMAPs is also limited. Since hydrophobic drugs tend to locate within the lipid bilayer as mentioned above, CsA-LVs have a great EE of 98.0 ± 2.8%, maximizing the drug dose in the CsA-LVs@DMAPs.

### CsA-LVs@DMAP characterisation

**Fig. 3:**
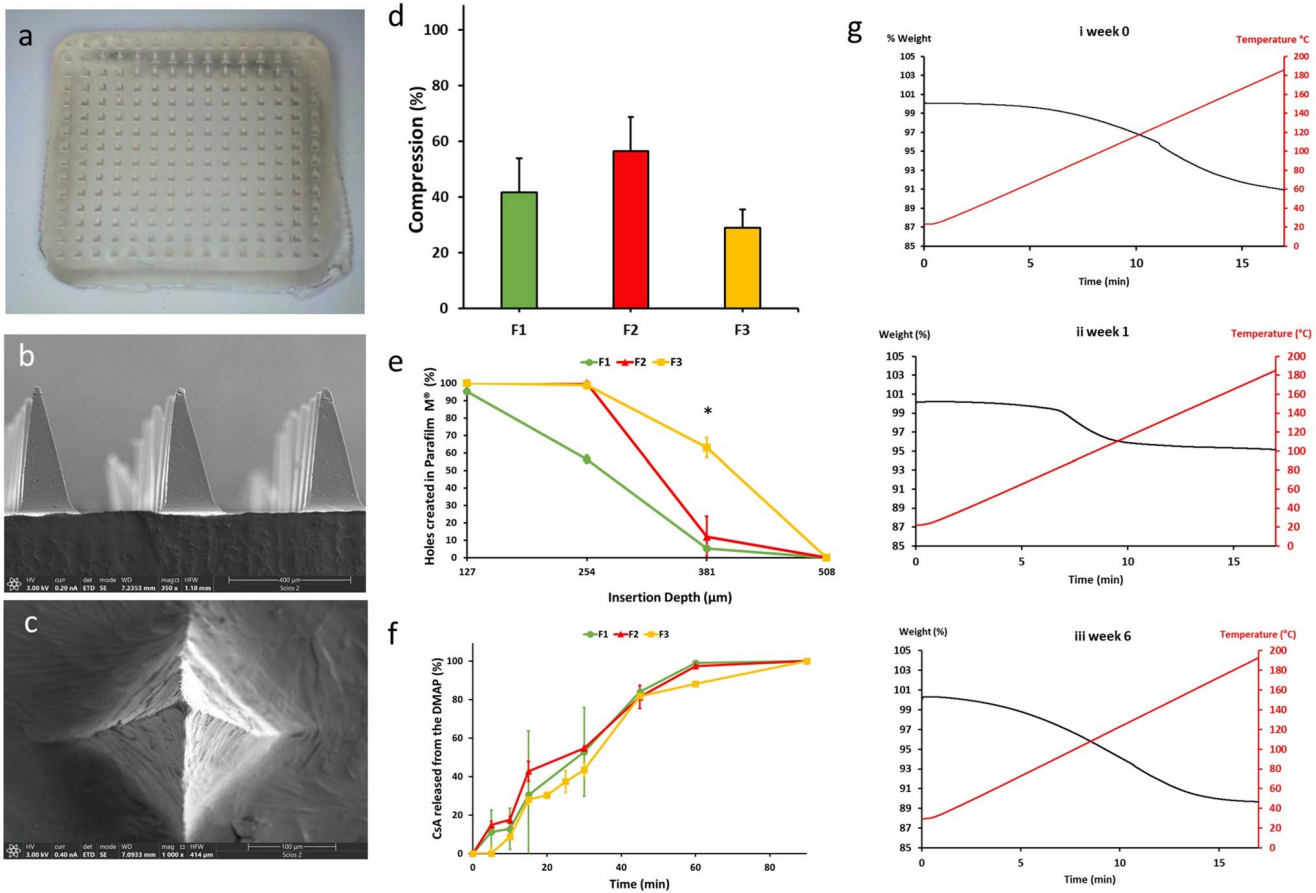
**a** Macroscopic image of CsA-LVs@DMAPs; **b, c** SEM images of the CsA-LVs@DMAPs. Scale bars: 400 µm (**b**), 100 µm (**c**); **d** compression percentage of the different DMAP prototypes. Results are expressed as mean ± SD (*n* = 3); **e** insertion percentage from different DMAP prototypes in Parafilm-M^®^ artificial skin model. Results are expressed as mean ± SD (*n* = 3); **f** release profile from different DMAP prototypes. Results are expressed as mean ± SD (*n* = 3); **g** TGA of the F3 formulation: (i) after fabrication, (ii) 1 week after fabrication and (iii) 6 weeks after fabrication. * denotes statistically significant differences in comparison with the other prototypes

DMAPs were successfully fabricated by the solvent-casting method and consisted of devices with 256 needle-like projections in a 16 × 16 needle arrangement (Fig. 3a). The morphology and length were observed by SEM imaging and optical microscopy (Fig. 3b, c), showing a pyramidal-sharp needle tip of 389 ± 5 µm. This needle length can penetrate the skin structure, without triggering a pain response, as shown by Kaushik et al., who proved a painless insertion with 550-µm needle tips [81].

Three different prototypes of DMAPs varying the PVP and PVA content were fabricated, and their mechanical properties were compared. The higher mechanical strength was observed in the prototype where PVA and PVP are combined (F3), with a deformation rate of 30 ± 7%, whereas compression of prototypes containing only PVA (F1) and PVP (F2) was 42 ± 12% and 57 ± 12%, respectively (Fig. 3d). This finding agrees with the reported literature, as it has been observed that the hydrogen-bond interactions between PVA hydroxyl groups and PVP carbonyl groups increase the mechanical properties and resistance of DMAPs [59]. This outcome is in correlation with the insertion performance. The insertion study demonstrated that all prototypes could penetrate deep enough to perforate the SC, whose thickness in human skin is around 20–30 µm [29]. However, F3 prototype was able to penetrate deeper into the artificial skin model, and its needle tips reached a depth of 381 µm in a proportion of 63 ± 12%. F1 and F2 prototype penetration values were significantly lower in comparison to F3 (*p* < 0.05), which only could reach 12 ± 11% and 5 ± 5%, respectively (Fig. 3e). The release study showed that all formulations were able to deliver the full load of CsA within 90 min (Fig. 3f). However, it should be noted that the in vitro model used does not mimic completely the in vivo conditions, where the interstitial fluid volume is much lower than the release media needed for sinking the DMAPs. Thus, the in vivo profiles can differ significantly, and it should be expected that the complete release of the cargo will take longer. Moreover, the F3 prototype, from now on referred to as CsA-LVs@DMAPs, showed the best properties over F1 and F2 which were not considered for the rest of the experiments. CsA-LV integrity was ensured based on their size, PDI and ζ-potential after dissolving the CsA-LVs@DMAPs in PBS pH 7.4. The obtained results were 158 ± 16 nm, 0.197 ± 0.057 and − 14.9 ± 0.7 mV (Fig. 2b, c), respectively. No differences were noticed after including the CsA-LVs in the DMAP structure in comparison with the initial characterisation properties (*p* > 0.05), thus proving that they remain stable throughout the manufacturing process and stay unaltered after their release.

DMAPs performance is often conditioned by environmental water absorption or insufficient drying process [82]. Residual water content was monitored by means of TGA. Representative thermograms of CsA-LVs@DMAPs are shown in Fig. 3g. It can be observed that the water content remained stable for 6 weeks below 4% when stored in a dry environment. Particularly, water levels were 3.60%, 3.14% and 3.39% after 48-h, 1-week, and 6-week fabrication, remaining below the recommended 10% [83]. Therefore, all the experiments done in this work were performed within this period, as CsA-LVs@DMAPs were not affected by hygroscopicity issues and remained stable, thus guaranteeing their mechanical properties.

### In vitro CsA-LVs@DMAP toxicity in fibroblasts and keratinocytes

Since the CsA-LVs and CsA-LVs@DMAPs developed in this study are intended to interact with skin cells, it was important to evaluate if they can induce cellular toxicity in skin cell lines. Therefore, the developed formulations were tested in L929 fibroblasts and HaCaT keratinocytes [84, 85]. The ATP content in metabolically active cells was measured by the CellTiter-Glo^®^ after 24 h of exposition to CsA-LVs and CsA-LVs@DMAPs, which was the expected time of exposition in the following in vivo DTH murine mode.

**Fig. 4:**
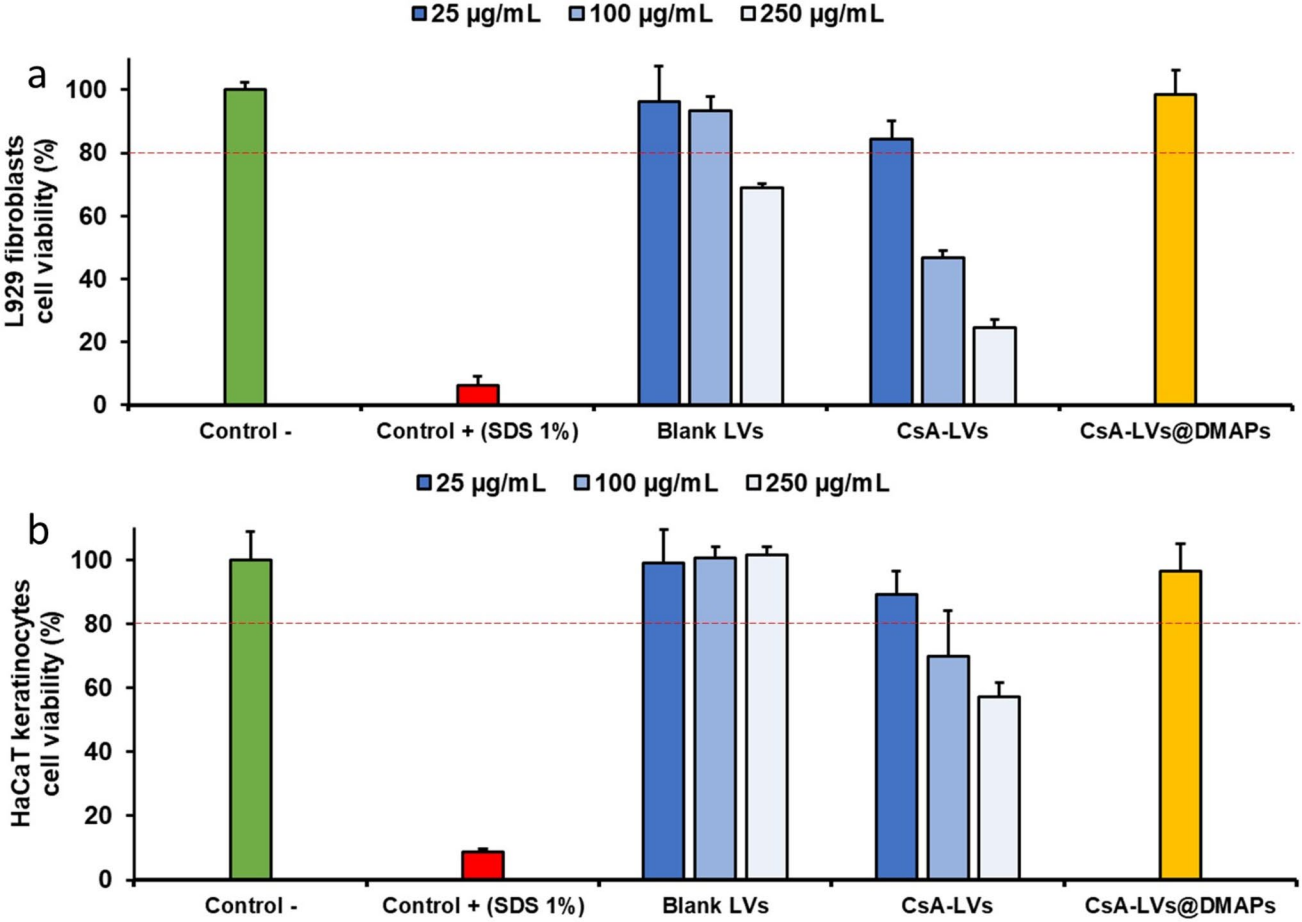
**a** L929 fibroblast cell viability after 24 h of treatment with CsA-LVs@DMAP components. Results expressed as mean (percentage) ± SD; **b** HaCaT keratinocyte cell viability after 24 h of treatment with CsA-LVs@DMAP components. Results expressed as mean (percentage) ± SD. Red line indicates 80% cell viability

As observed in Fig. 4a, b, L292 fibroblast showed a higher sensitivity to the liposomal formulations rather than HaCaT keratinocytes. Particularly, blank LVs were only toxic in L292 fibroblast when exposed to a 250 µg/mL concentration (Fig. 4b). Moreover, when LVs were loaded with CsA, a higher toxicity was noticed because of the potential side effects of the drug on the cells. he CsA-LVs showed moderate and weak cytotoxicity at 100 and 250 µg/mL concentrations in keratinocytes and fibroblasts, respectively. However, these concentrations are much higher than the expected levels after their administration, and they showed acceptable cell viability above 80% at 25 µg/mL after 24 h. PVA and PVP are polymers with excellent biocompatibility that has been proved in several studies [86, 87], so their combination with CsA-LVs showed optimal compatibility with both cell lines.

### Ex vivo CsA-LVs@DMAP skin insertion performance, dissolution time and drug retention

**Fig. 5:**
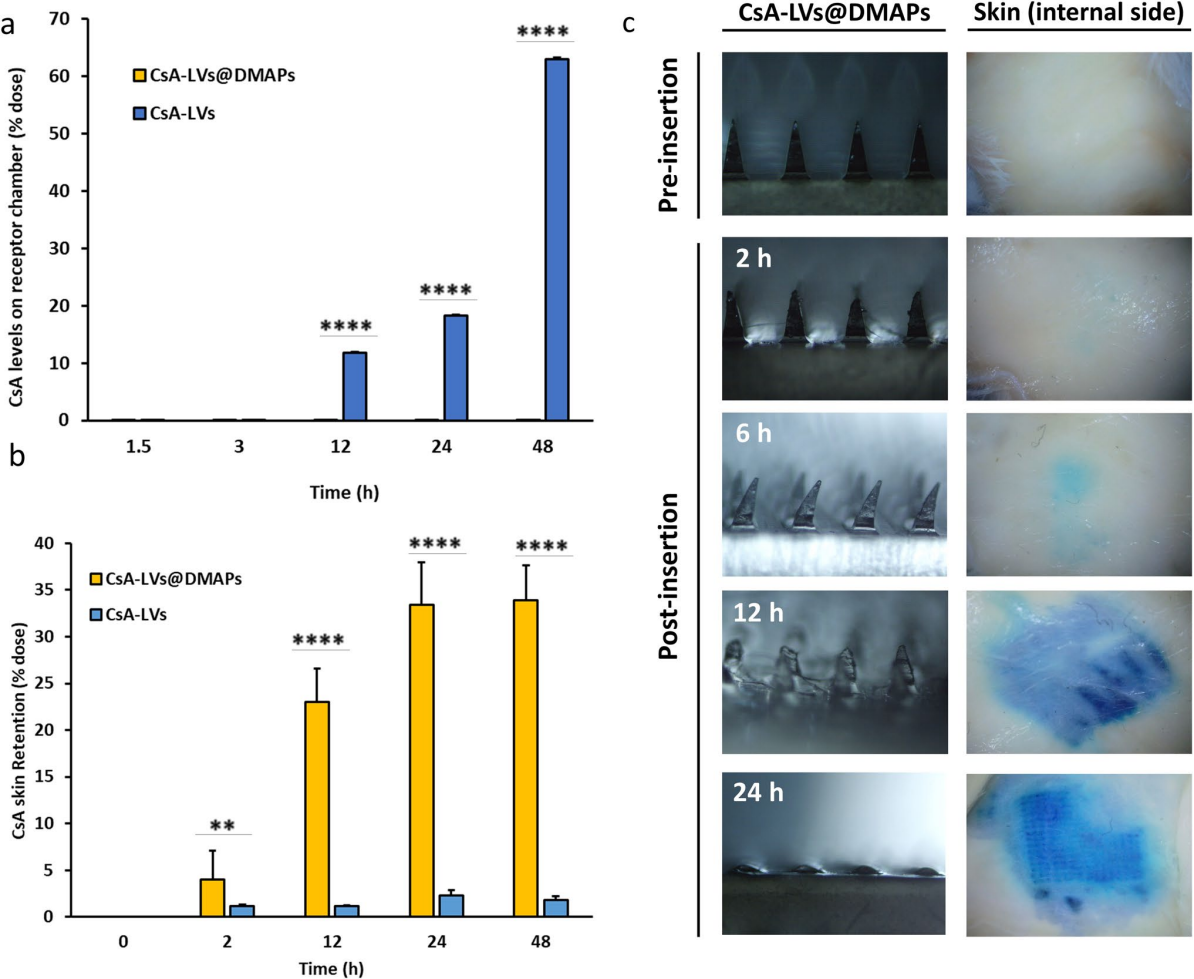
**a** Cumulative ex vivo amounts of CsA found in the receptor FDC chamber after 1.5, 3, 12, 24 and 48 h. Results expressed as mean (percentage of initial dose) ± SD (*n* = 5); **b** Cumulative amount of CsA found in the skin structure after drug extraction at 2, 12, 24 and 48 h (*n* = 3). Results expressed as mean (percentage of initial dose) ± SD. ** and **** denote significant differences between the comparison pair (*p* < 0.01 and *p* < 0.0001, respectively); **c** representative pictures pre-insertion and post post-insertion of CsA-LVs@DMAPs and skin staining (internal side) after CsA-LVs@DMAP dissolution

Although some studies have previously administered CsA through the skin using emulsions [88], the percutaneous administration of the molecule is still limited. Carreras et al. [33], Silva et al. [89], Essaghraoui et al. [90] and Wairkar et al. [91] have used lipid-based systems to deliver CsA transdermally. However, the main disadvantage of these strategies is that they predict or allow CsA access to the peripheral circulation, increasing its side effect risks. In correlation with those studies, the CsA-LV dispersion was also capable of delivering CsA to the donor chamber of the FDC in high amounts—specifically, 11 ± 3% of the initial dose at 12 h, 18 ± 3% at 24 h and reaching its maximum at 48 h, achieving 63 ± 7% from the initial dose—. The combination of LVs and solid MAPs boosts the permeability of the drugs, as demonstrated previously [92]. However, the combination of LVs and DMAPs can provide an extra controlled release of the cargo, and therefore, when CsA-LVs@DMAP permeability was examined, CsA was not found in the receptor media (Fig. 5a), avoiding the appearance of systemic off-target effects [93–96].

Since the CsA-LVs@DMAPs were prepared in a one-step manufacturing protocol, the CsA accumulation in the skin structure was also studied to determine the exact dose of CsA that is delivered by CsA-LVs@DMAPs in the skin (Fig. 5b). On the one hand, skin treated with CsA-LVs@DMAPs accumulated 13.38 ± 1.76 µg CsA after 24 h, representing the 33 ± 5% of the total dose. This amount does not differ from the 13.56 ± 1.49 µg of CsA after 48 h (34 ± 4% of the initial dose). On the other hand, in skin treated with CsA-LVs, the drug levels were lower (around 2% from the initial dose). These findings demonstrate that the maximum release from CsA-LVs@DMAPs is achieved after 24 h from the insertion and highlight that CsA-LVs@DMAPs provide a higher bioavailability of CsA in skin structure, which is crucial for maximizing their potential efficacy to alleviate the skin-related conditions.

The CsA-LVs@DMAP ability to penetrate the skin structure and the dissolution time needed to release the cargo were additionally assessed in an ex vivo setup using full-thickness mice excised skin. Figure 5c shows the time-dependent tip dissolution after the insertion into the skin. The images correlate with the findings observed in the ex vivo skin retention studies, where the maximum drug release was obtained after 24 h. Specifically, CsA-LVs@DMAPs barely dissolve during the initial post-insertion hours, and after 6 h, they only slightly bend. Although in ex vivo retention studies, it was possible to detect CsA release after 2 h, the co-loaded blue dye did not stain the internal side (deeper skin layers) of the skin, thus confirming that the dissolution process of the DMAPs remains in an early phase. Only after 12 h an evident dissolution takes place, reaching the full dissolution of the CsA-LVs@DMAP tips 24 h after their insertion.

### In vivo DTH murine model of skin inflammation

**Fig. 6:**
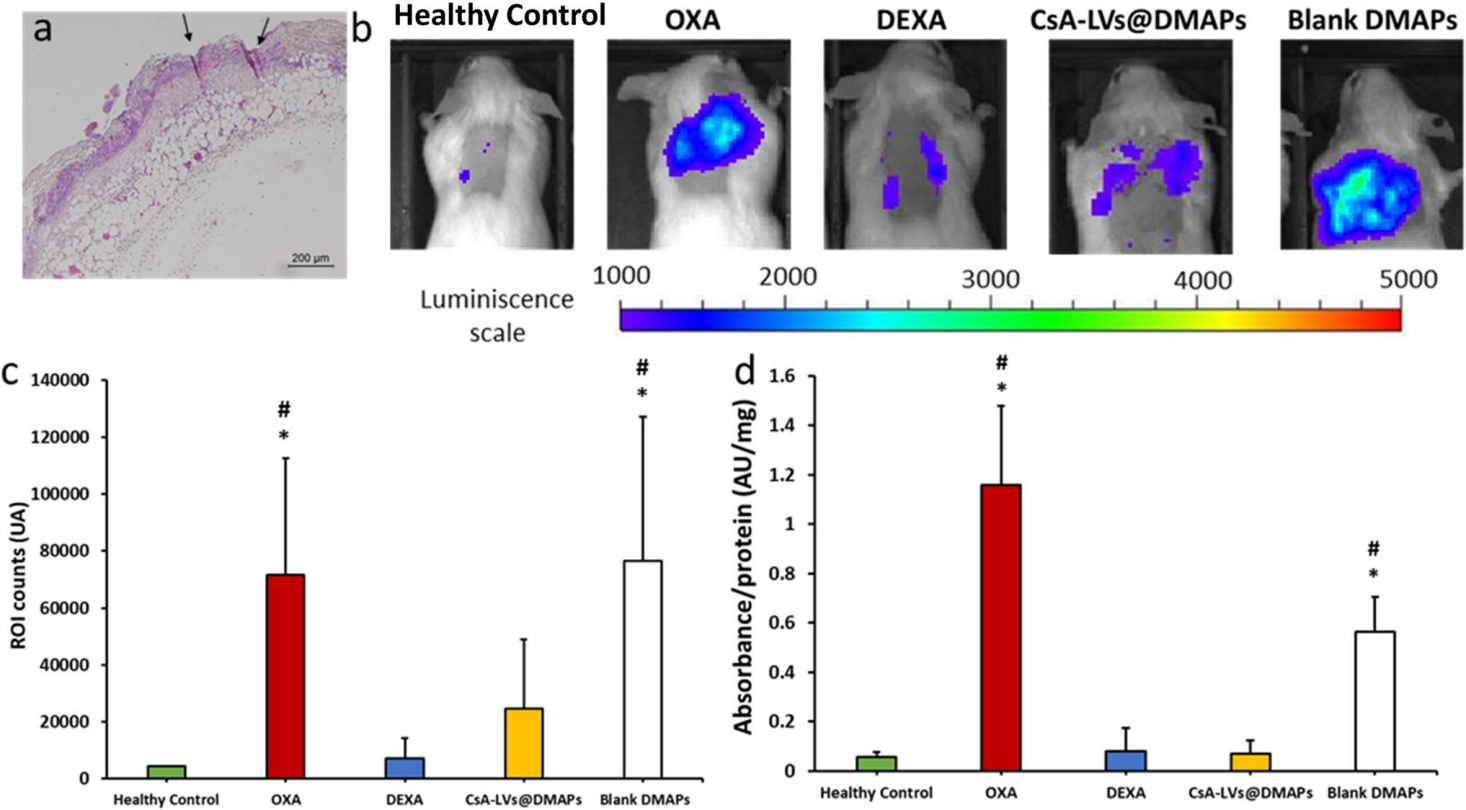
**a** Representative histology image CsA-LVs@DMAP insertion in the DTH murine in vivo model. Arrows indicate the place where DMAPs were inserted exactly; **b** representative images of BLI luminescence 24 h after OXA challenge of the different groups involved in the experiment: Healthy control (unchallenged), dexamethasone-treated group (DEXA), CsA-LVs@DMAP-treated group, non-treated group (OXA) and blank DMAP–treated group. Scale was set up from 200 up to 4000 photons;** c** ROI counts of BLI induced after the different treatments. ROI area was in the exact application place of the DMAPs and set equal for each subject under study. Results are expressed as mean ± SD (*n* = 6); **d** MPO quantification in skin tissue after the different treatments. MPO activity was expressed as absorbance/mg of protein. Results are expressed as mean ± SD (*n* = 3). * denotes statistically significant differences in comparison to healthy control (*p* < 0.05). # denotes statistically significant differences in comparison to the DEXA group (*p* < 0.05)

The pivotal aim of this work was to study the effectiveness of the developed CsA-LVs@DMAPs in an in vivo murine DTH model, which has long been recognised and standardised as a skin inflammation and dermatitis-like model. For that, mice challenged with OXA (after a previous sensitization stage) experienced an inflammatory response which involves neutrophils, macrophages and T cells at the cellular level [97]. CsA-LVs@DMAP performance in ameliorating skin inflammation was evaluated by means of BLI, MPO activity, histological damage evaluation and pro-inflammatory cytokine quantification. To the best of our knowledge, this is also the first time that the DTH model has been challenged with a DMAP-assisted technology. Although in the previous ex vivo studies, it was confirmed that CsA-LVs@DMAPs can penetrate the skin and improve drug retention in the skin structure, the successful insertion under in vivo conditions was confirmed by a histological inspection. It can be observed the effective micro-channels creation after the CsA-LVs@DMAP application (Fig. 6a). BLI was used for live assessment of inflammation, since BLI is an indirect measurement of MPO activity that is strictly related to neutrophils and macrophages activity and their infiltration in the affected tissue [98–100]. BLI was measured 24 h after the OXA challenge, and representative images of photon emission are shown in Fig. 6b. OXA clearly triggered the photon emission (OXA group), which was normalised after the treatment with a TCS-like treatment (DEXA group). When compared to the OXA group, CsA-LVs@DMAP-treated animals showed a decreased bioluminescence intensity, whereas blank DMAPs showed a slight increase of photon emission intensity. The photon counts were quantified in the exact insertion area of DMAPs (Fig. 6c). The OXA group exhibited a significantly higher signal in comparison to the healthy (unchallenged) control group, proving that in this DTH model, OXA triggers an intense inflammatory reaction (*p* < 0.05). As expected, ROI counts in the OXA group were also normalised after DEXA treatment (*p* < 0.05); thus, the suitability of the model was confirmed, since OXA pathological effects were prevented by topical application of this TCS. ROI counts in CsA-LVs@DMAP-treated mice were similar in comparison to the healthy control and DEXA groups (*p* > 0.05) and significantly lower than the OXA group (*p* < 0.05). Consequently, the anti-inflammatory performance of the CsA-LVs@DMAPs was comparable to DEXA results and could successfully revert the OXA challenge. Remarkably, blank DMAPs showed an even stronger photon emission than OXA.

Although some authors report no inflammation triggered by DMAPs [101], in this experiment, to guarantee DMAP attachment for 24 h and avoid their removal by the animals, they were secured to the skin using stitches, which can contribute to the increase of inflammation in the wounded skin. The use of stitches was exclusively used to secure completely the CsA-LVs@DMAPs for the intended application time, and it could explain why a BLI signal is observed in the surrounding areas of DMAP insertion for the CsA-LVs@DMAP group (Fig. 6b). However, stitches would not be needed if DMAPs are applicated in clinics. Besides, the creation of pores through the needles can enhance the penetration of OXA, compared to non-treated skin, thus increasing its inflammatory properties in the blank DMAP group. The trend observed in BLI was corroborated by the direct MPO quantification from skin homogenates (Fig. 6d). Once again the DEXA, healthy control and CsA-LVs@DMAP groups showed similar activity of this enzyme, and no statistical differences were found between groups (*p* > 0.05), while the OXA and Blank DMAP groups showed upregulated MPO levels (*p* < 0.05).

**Fig. 7:**
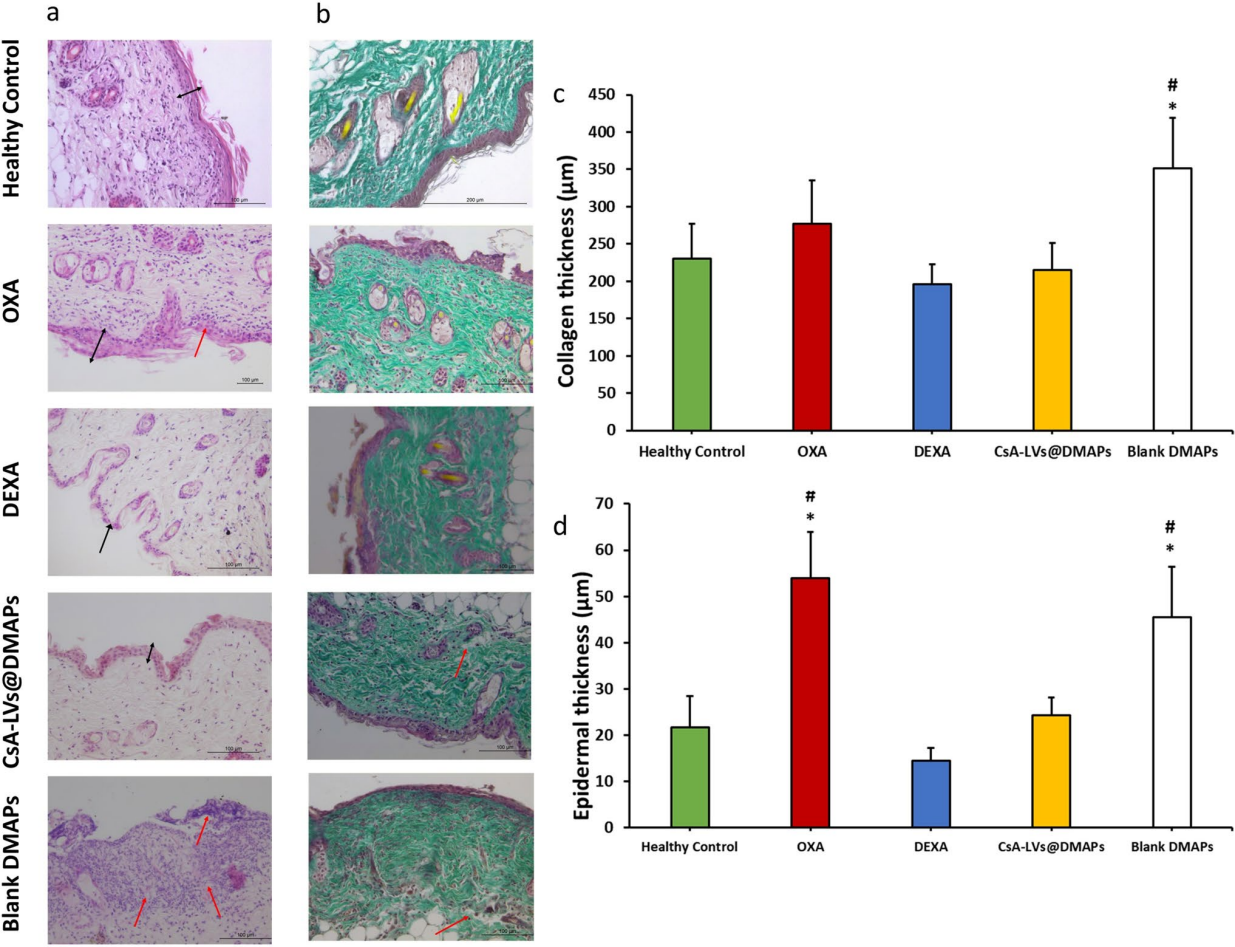
**a** Representative images from the histological sections stained with H&E stain (× 20 magnifications); **b** representative images from the histological sections stained with MT stain (× 20 magnifications); black arrows indicate the epidermal thickness, and red arrows indicate spongiotic patterns and neutrophile infiltration. **c** Collagen thickness measured from histological observation 24 h after the administration of the different treatments on the MT stains at × 10 magnifications; **d** epidermal thickness measured from histological observation 24 h after the administration of the different treatment H&E stains at × 20 magnifications. Results are expressed as mean ± SD (*n* = 3); results are expressed as mean ± SD (*n* = 3). * denotes statistically significant differences in comparison to healthy control (*p* < 0.05). # denotes statistically significant differences in comparison to the DEXA group (*p* < 0.05)

Histopathological damage was also assessed by the anatomical differences observed in the H&E stain (Fig. 7a). On one side, no notable differences between the DEXA and CsA-LVs@DMAP groups were found in comparison with the healthy control group. On the other side, a higher leukocyte infiltration was observed in the OXA and blank DMAP groups, which is considerably lower in DEXA and CsA-LVs@DMAPs. Moreover, spongiotic patterns and abscess were also detected in the OXA and blank DMAP groups, suggesting acute inflammatory damage. Collagen is an indicator of dermis thickness, and its formation is upregulated in inflammatory skin diseases such as contact and atopic dermatitis [102]. Collagen thickness was determined by MT stain measuring the green-blue stained areas (Fig. 7b). In Fig, 7c, it can be observed that a non-significant increase of collagen thickness is noticed in the OXA group compared to the healthy control (*p* > 0.05). However, as described in other studies working with this model, the significant differences in fibrotic events can be appreciated after 24 h but are more evident at 72 h post-challenge [103]. Moreover, the combination of OXA challenge and blank DMAP insertion increased collagen deposition, probably due to granulation tissue formation after the wounds inflicted by the stitches. This increase of collagen fibres was not observed in CsA-LVs@DMAPs (*p* > 0.05), suggesting again the anti-inflammatory effects of CsA. Similarly, DEXA treatment also showed a reduced collagen thickness, as a consequence of the skin atrophy related to the use of TCS probably [104]. A similar trend was also observed when epidermal thickness was measured in H&E stain (Fig. 7a). As expected, the epidermis was thicker in the OXA and blank DMAP group (*p* < 0.05), as observed in Fig. 7d. Favourably, CsA-LVs@DMAPs reduced the epidermal thickness regarding the OXA and blank DMAP group, while no differences were found between the healthy control and DEXA groups (*p* > 0.05).

**Fig. 8:**
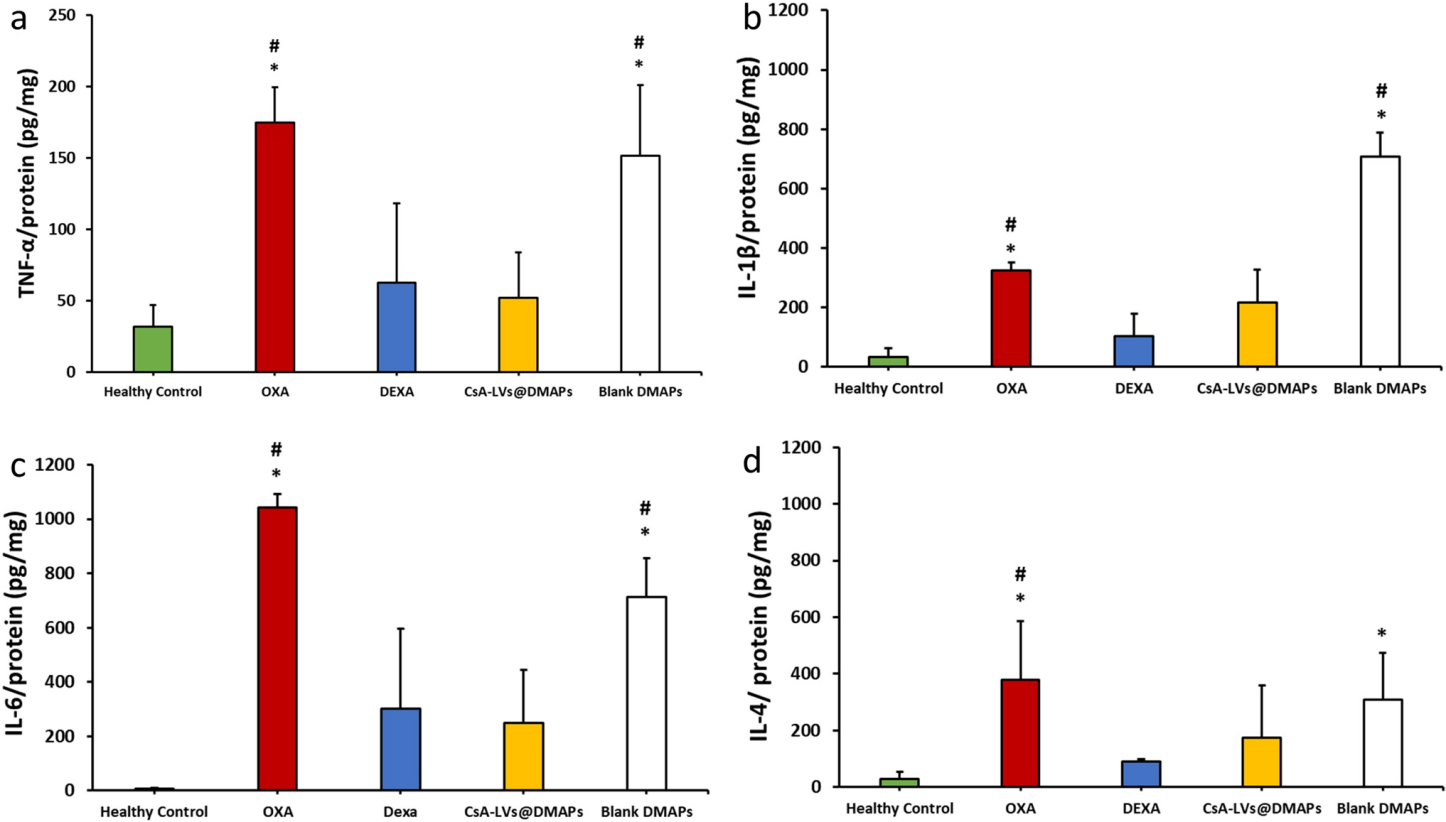
**a** TNF-α quantification detected by ELISA immunoassay in skin samples; **b** IL-6 quantification detected by ELISA immunoassay in skin samples; **c** IL-4 quantification detected by ELISA immunoassay in skin samples; **d** IL-1β quantification detected by ELISA immunoassay in skin samples. Results are expressed as mean (pg of cytokine/mg of protein) ± SD (*n* = 3). * denotes statistically significant differences in comparison to healthy control (*p* < 0.05). # denotes statistically significant differences in comparison to the DEXA group (*p* < 0.05)

TNF-α, IL-6, IL-4 and IL-1β cytokines were determined as inflammatory markers for skin inflammation (Fig. 8a–d). High levels of these proteins are related to a pro-inflammatory environment. Specifically, IL-1β is upregulated in contact and acute dermatitis [105], while high levels of IL-6 are strongly related to the immune component of these diseases and skin damage [106]. In addition, TNF-α elicits a cascade amplification effect in dermatitis [107], whereas IL-4 plays an important role in type-II inflammation pathway and dermatitis-induced skin lesions [108]. In general, the cytokine detection experiments showed the same trend, and no significant differences were observed when comparing CsA-LVs@DMAPs with the healthy control and DEXA groups (*p* > 0.05), pointing out the effectivity of CsA-LVs@DMAPs for reducing the inflammation state after the OXA challenge. However, it can be appreciated that the OXA and blank DMAP groups showed a significant increase of the pro-inflammatory cytokines (*p* < 0.05). Overall, all the findings compiled from this in vivo DTH murine model support the success of CsA-LVs@DMAPs in relieving the signs and symptoms of skin inflammation, typically observed in contact and atopic dermatitis conditions.

Finally, blood samples were taken from the CsA-LVs@DMAP group at 24 h and 48 h to measure the CsA plasma levels (*n* = 3). Interestingly, no levels of CsA were found as predicted from the ex vivo permeability study (Fig. 5a), although there is a possibility that small amounts of CsA were cleared within 24–48 h or even after. Hence, the formulation does not show any evidence for subsequent developing CsA-associated off-target effects, as the drug did not access the systemic circulation, at least above the analytical detection limit.

Overall, this work aims to contribute to the ever-growing body of evidence on the effectiveness of microneedle systems for drug delivery. Although DMAPs are ideal for deployment in low-resource areas, manufacturing processes of DMAPs are important challenges in fabrication and scalability that have slowed their development. However, mass production of DMAP devices has benefited from the revolution of 3D printers and related technologies easily adaptable to the solvent-casting production method that has made microneedle-based technology a real alternative to current treatments [109]. Nevertheless, clinical translation is a hurdle that microneedle-based delivery systems are overcoming currently. The US FDA has expressed concerns about the quality of submissions involving combination products utilising microneedles, particularly focusing on stability testing, ensuring content consistency, conducting a thorough risk analysis, validating sterility and maintaining high standards in the manufacturing and storage process [110]. The issues related to stability, safety and sterility concerns seem to be solved since numerous works reported that polymer-based DMAP products keep their properties upon use without big preserving measures [111] and do not produce evident toxic effects in long-term treatments [86], and although DMAPs can be altered by the application of dry heat or gamma-radiation [112], they remain stable after several techniques such as ethylene oxide and electron beam sterilisation [113, 114]. Despite that DMAPs can be successfully inserted into the skin with minimum training using thumb force or commercially available applicators, self-administration by patients is a double-sword, since they might not receive clear feedback beyond the macroscopic observation of DMAPs. Therefore, the microneedle-based field still evolving to fulfil this limitation and rapid-separable tips have already been designed to facilitate the correct administration [51].

## Conclusion

CsA-LVs@DMAPs are a suitable nano-in-micro device to achieve CsA skin permeability and enhance its retention in the skin structure. CsA-LVs allowed the loading of CsA into the DMAP matrix despite its hydrophobic physicochemical properties. The CsA-LVs@DMAP characterisation showed effective skin penetration, good physical stability after 6-week storage and an improvement of ex vivo dose retention within the skin structure in comparison to a transdermal of CsA-LV administration. In vitro experiments ensured the biocompatibility of CsA-LVs@DMAP formulation in representative skin cell lines. In vivo study carried out in a delayed-type hypersensitivity murine model of dermatitis confirmed the efficacy of CsA-LVs@DMAPs to ameliorate and relieve the skin-associated inflammation. Specifically, bioluminescence signals, skin and collagen thickness, myeloperoxidase activity, histological damage and inflammatory cytokine levels were normalised after the CsA-LVs@DMAP application, showing a similar efficacy than a dexamethasone reference treatment. Furthermore, CsA plasmatic levels were not detected after the CsA-LVs@DMAP application, guaranteeing the absence of CsA systemic absorption and avoiding eventually its systemic side effects. All these results point out that CsA-LVs@DMAPs are a feasible alternative to topical corticosteroids treatment, in order to minimise their associated side effects especially in long-term and chronic treatments.

## Data Availability

The data that support the findings of this study are available from the corresponding author upon reasonable request.
